# Recent Advances in Porous Polymer-Based Flexible Piezoresistive Pressure Sensors

**DOI:** 10.3390/polym17192584

**Published:** 2025-09-24

**Authors:** Junwei Huang, Zhongxin Xu, Jing Zhang, Yujun Wei, Bo Peng, Guanwei Liang, Shudong Yu

**Affiliations:** 1 School of Advanced Manufacturing, Sun Yat-sen University, Shenzhen 518107, China; 2 School of Integrated Circuits, Sun Yat-sen University, Shenzhen 518107, China; 3 School of Fashion and Textiles, The Hong Kong Polytechnic University, Hung Hom, Kowloon, Hong Kong 999077, China; guanwei.liang@polyu.edu.hk

**Keywords:** porous polymer, conductive filler, flexible pressure sensors, piezoresistive effect, wearable devices

## Abstract

With the rapid development of wearable devices and intelligent human–machine interaction technologies, the demand for high-precision pressure sensors has soared. Piezoresistive pressure sensors excel due to their simple structure, low cost, and high sensitivity, among which flexible piezoresistive pressure sensors based on porous polymers have become a research focus, thanks to their unique 3D porous structure and excellent performance. This review summarizes recent advances: it introduces key performance metrics and the piezoresistive sensing mechanism; outlines porous structure preparation methods (phase separation, 3D printing, electrospinning) with their principles, advantages, and limitations; examines conductive fillers (carbon-based, polymer, metal, MXene) with their properties and applications; and highlights flexible substrates (silicone, polyurethane, polyimide, natural polymers) in ensuring mechanical compliance and device integration. Studies show material innovation, structural optimization, and process improvement can significantly enhance sensor accuracy, stability, and durability, helping break traditional performance bottlenecks. Future prospects are broad in tactile sensing, biomedical monitoring, and human–machine interaction, providing references for related research and industrial development.

## 1. Introduction

**Figure 1:**
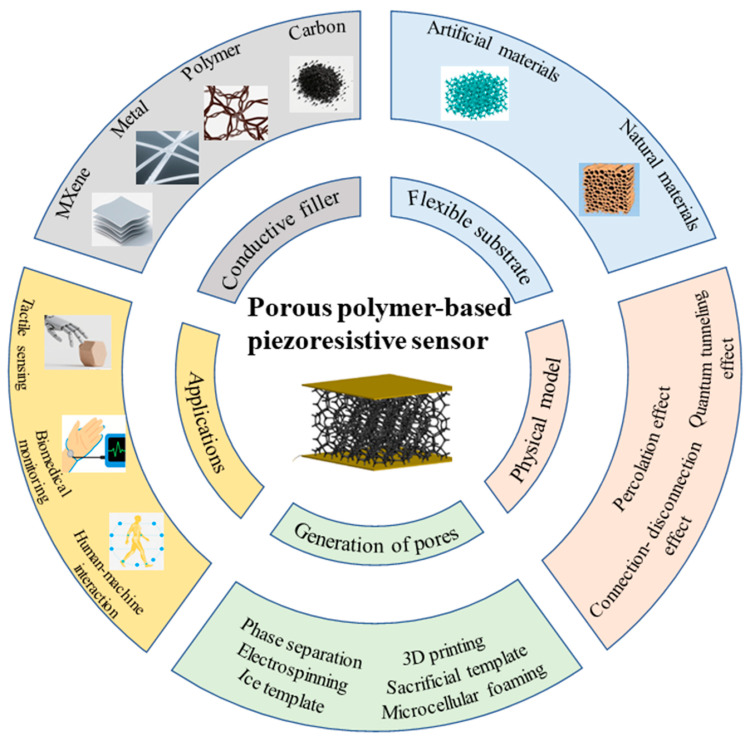
Porous polymer-based flexible piezoresistive pressure sensors: selection of flexible substrates based on polymer porous materials, conductive fillers, fabrication methods of porous structures, physical models, and applications. Adapted with permission from Ref. [1]. Copyright 2022, Elsevier and adapted with permission from Ref. [2]. Copyright 2021, Elsevier.

Against the backdrop of rapid advancements in piezoresistive sensing technologies and the expanding applications of porous polymers, this article is presented to comprehensively synthesize the relevant research progress. Specifically, it reviews the research progress of flexible piezoresistive pressure sensors based on porous polymer composites, outlines evaluation criteria and sensing mechanisms for this type of sensor, details the manufacturing methods for porous structures, and systematically introduces the conductive fillers and flexible substrates used in sensor fabrication, along with the application domains of these sensors. Finally, the paper provides a summary of the current development status in this field and anticipates future research directions. Figure 1 provides a brief summary of the work in this article.

**Figure 2:**
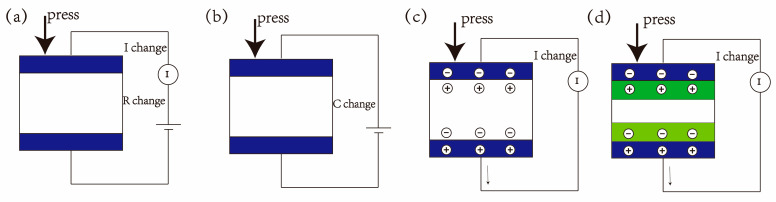
Sensing mechanisms of (**a**) piezoresistive sensor, (**b**) capacitive sensor, and (**c**) piezoelectric sensor, (**d**) triboelectric sensors.

In the current era of rapid technological development, the rise in wearable electronic devices and intelligent systems have led to an increasing demand for sensors capable of accurately perceiving physical stimuli. Pressure sensors, which can be used for human motion detection [3,4,5], physiological signal sensing [6,7,8], and intelligent electronic skin [9,10], have become a research hotspot. The main working principle of a pressure sensor is to receive pressure signals and convert them into electrical signals under external loads. Pressure sensors can be divided into four types: piezoresistive [11], capacitive [12], piezoelectric [13], and triboelectric [14] sensors. While piezoresistive and capacitive sensors require an external power supply to generate signals, piezoelectric and triboelectric sensors can autonomously generate charges and produce signals without the need for an external power source. Schematic diagrams illustrating the sensing mechanisms of these four sensor types are shown in Figure 2. Compared to capacitive, piezoelectric and triboelectric sensors, piezoresistive pressure sensors offer several advantages: they exhibit high sensitivity over a wide pressure range, excellent linearity and stability, and their output signals are easy to process and calibrate. Furthermore, piezoresistive sensors demonstrate fast response, allowing for the rapid detection of pressure changes and fulfilling real-time monitoring requirements [15].

Among the various types of flexible piezoresistive sensors, those based on porous polymers stand out. The unique three-dimensional porous structure of porous polymers imparts a range of exceptional properties to the sensors. On one hand, the porous structure provides a large specific surface area, which not only enhances the contact area with the external environment, improving the sensor’s ability to detect physical stimuli, but also facilitates the dispersion of conductive fillers, thereby promoting the formation of a stable conductive network. On the other hand, the pore structure bestows good flexibility and compressibility to the polymer. When subjected to external forces, the pores undergo reversible deformation, which in turn leads to changes in resistance, enabling accurate measurement of pressure and strain.

Flexible piezoresistive sensors based on porous polymer composites are primarily composed of a flexible substrate and conductive fillers. Common polymer substrate materials that can easily form porous structures through conventional processing techniques include polydimethylsiloxane (PDMS) [16,17,18], polyurethane (PU) [19,20,21], polyimide (PI) [22,23], and cellulose. Common conductive fillers include carbon-based conductors [24,25], conductive polymers [26], metal conductors [27], and MXene [28]. The flexible substrate typically serves to support the load, while the conductive filler not only imparts the conductivity of the composite material but also improves the mechanical properties of the flexible substrate and boosts the response sensitivity.

## 2. Performance Metrics and Physical Mechanism of Piezoresistive Sensing

### 2.1. Performance Metrics

Based on the currently reported research on flexible piezoresistive sensors based on porous polymer materials, the following metrics are generally considered key parameters for evaluating their performance.

#### 2.1.1. Sensitivity

Sensitivity is a key metric for measuring the ability of a sensor to respond to external disturbances. For pressure sensors, sensitivity is usually defined as the ratio of the relative change rate of resistance to the change in applied pressure, and the formula is

(1) $$S=\frac{∆R/R_{0}}{∆P}$$

where Δ*R* represents the difference between the resistance after being under pressure and the resistance without pressure, *R*_0_ represents the original resistance, Δ*P* represents the pressure, and the unit of sensitivity *S* is kPa^−1^.

When a pressure sensor is used to perceive strain, the gauge factor (*GF*) is often used to reflect its performance, and the formula is

(2) $$GF=\frac{∆R/R_{0}}{\varepsilon }$$

where *ε* represents the strain, defined as the ratio of the change in length to the original length.

#### 2.1.2. Linearity

Linearity characterizes the degree of linear correlation between the sensor’s output signal (typically Δ*R/R*_0_) and the input pressure. It is usually quantified by the deviation between the fitted straight line and the measured data (nonlinear error) or by the correlation coefficient (R^2^). High linearity simplifies the signal calibration process and enhances measurement accuracy, which is especially important in applications involving a wide pressure range. However, porous polymers tend to experience plastic collapse or pore saturation under high pressure, leading to a piecewise linear resistance-pressure response. Typically, the slope is steep in the low-pressure region and shallow in the high-pressure region, necessitating the use of different functions for different pressure ranges, which can introduce errors in pressure measurement. Incorporating a gradient pore distribution into the porous structure design or combining it with a high-elasticity microstructure can effectively mitigate the issue of plastic collapse under high pressure, thereby improving the sensor’s linear response. Recently, researchers have successfully achieved a wide linear detection range at the MPa level [29].

#### 2.1.3. Response/Recovery Time

Response time and recovery time together constitute a key dimension for evaluating the dynamic performance of the sensor. A short response time ensures that the sensor can instantly capture transient pressure events, such as the pulsation of the heartbeat or the rapid movement of joints, avoiding the loss or delay of key information. Recovery time determines whether the sensor can quickly return to its initial state after the pressure is removed, providing a guarantee for continuous and real-time monitoring. These two parameters directly reflect the ability of the sensor to track dynamic signals, and their performance is mainly limited by the viscoelasticity of the polymer material, the compression and rebound characteristics of the porous structure, and the reconstruction speed of the internal conductive network under deformation.

#### 2.1.4. Long-Term Stability

Long-term stability reflects the long-term reliability and durability of the sensor. It characterizes the ability of the sensor to maintain its core performance (such as sensitivity) without attenuation after undergoing repeated loading-unloading cycles. In practical operation, the number of cycles that maintain the original performance, i.e., fatigue life, is used as an evaluation metric. Excellent fatigue resistance is crucial for scenarios that require long-term and frequent use, such as continuous health status monitoring, online condition monitoring of industrial equipment, or daily interaction with wearable electronic devices. Improving fatigue life depends on the selection of flexible substrate materials with high resilience and low creep properties, the design of a robust porous structure that can resist plastic collapse and structural degradation and ensuring a firm and long-lasting interface bond between the conductive filler and the polymer matrix.

#### 2.1.5. Detection Limit

The detection limit is the minimum measurable pressure value that the sensor can reliably identify. This parameter directly determines the ability of the sensor to detect weak physical stimuli (such as microvascular pulsation and gentle touch). The advantage of a low detection limit of porous polymer piezoresistive sensors mainly comes from the large specific surface area provided by their high porosity structure and the excellent sensitivity of the conductive network, that is, a small pressure can cause significant pore deformation and conductive path reconstruction. Recent studies have successfully reduced the detection limit to 3.4 Pa [30]. Breaking through the bottleneck of the low detection limit is a key prerequisite for achieving accurate perception of small pressures.

### 2.2. Physical Mechanism of Piezoresistive Effect

The piezoresistive response of porous polymer composites emerges across three complementary levels. First, a static percolation framework set by filler concentration determines whether a continuous conductive network exists and where the operating point lies relative to the percolation threshold. Second, under mechanical loading, a dynamic percolation (connection–disconnection) process reconfigures the network, modulating the number and geometry of conductive pathways. Finally, at the nanoscale, quantum tunneling governs electron transport across residual gaps, especially when contacts saturate or local separations approach sub-nanometer dimensions.

**Figure 3:**
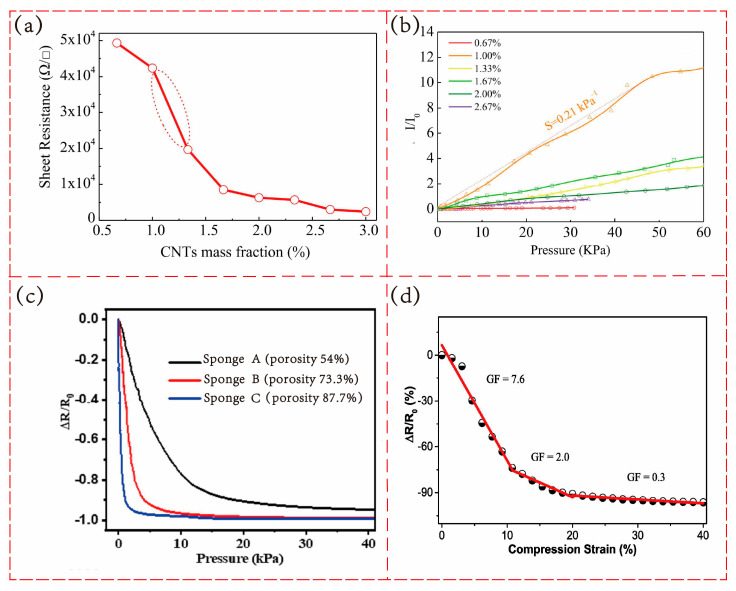
(**a**) The resistance of CNT/PDMS composite materials changes with the variation in CNT mass fraction. Reprinted with permission from Ref. [32]. Copyright 2022, Elsevier. (**b**) The sensitivity of sensors with different CNT mass fractions. Adapted with permission from Ref. [32]. Copyright 2022, Elsevier. (**c**) Piezoresistive transfer function under different porosities. Adapted with permission from Ref. [30]. Copyright 2024, Elsevier. (**d**) The transfer function of the sensor when it is jointly governed by the connection–disconnection effect and the tunneling effect. Adapted with permission from Ref. [18]. Copyright 2020, Elsevier.

Percolation effect: when conductive fillers are introduced into an insulating polymer matrix, a certain concentration must be reached to form a stable conductive path, which is referred to as the percolation threshold [31]. Once this concentration is exceeded, the resistivity of the composite material will sharply decrease with the increasing concentration of conductive fillers, which may lead to a reduction in sensor sensitivity. When the concentration of conductive fillers is below this value, it is impossible to form a stable conductive path, leading to poor electrical performance of the sensor. Therefore, a sensor with good sensitivity should operate in the range near the percolation threshold. It is necessary to continuously adjust the concentration of conductive fillers during the research process to achieve optimal sensitivity. Taking CNT/PDMS porous materials as an example [32], as shown in Figure 3a, when the mass fraction of CNT material is 1%, the composite material exhibits the maximum rate of resistance change. 1% can be approximated as the percolation threshold of the composite materials in this study. As shown in Figure 3b, the composite material with 1% CNT content has the best sensitivity, while sensors with slightly higher than 1% content also exhibit high sensitivity.

The percolation threshold often varies with the choice of materials; moreover, differences in composite fabrication methods also influence this threshold, making it complex to directly generalize the percolation threshold for a specific class of materials. Therefore, in practical fabrication of porous polymer-based flexible piezoresistive sensors, a common approach is to systematically vary the concentration of conductive fillers for comparative testing, thereby identifying a “champion” sensor with an optimal sensitivity [18,32,33]. It should be noted that all sensors discussed in this study operate above the percolation threshold.

Connection-disconnection effect: when the sensor is not under load, the conductive fillers form a network inside or on the surface of the flexible substrate, overlapping and connecting with each other. During the loading and unloading process, the structure of the conductive network may change, resulting in a change in the length or number of conductive paths, thereby causing a change in resistance. In most flexible piezoresistive sensors, when the sensor is under pressure, the conductive fillers touch closely with each other, thereby increasing the number of conductive paths and reducing the resistance of the material. When the pressure is released, the conductive fillers separate from each other, and the resistance increases. However, for some sensor models with microcracks [34], the expansion of cracks when under pressure leads to an increase in resistance, and the healing of cracks when unloaded leads to a decrease in resistance. The connection–disconnection mechanism is strongly modulated by the porosity of the polymer matrix. Guo and colleagues [30] evaluated the piezoresistive performance of MXene/PDMS composite sponges with different porosities. As shown in Figure 3c, the sponge with a porosity of 87.7% displayed the steepest slope in the resistance–pressure curve at low pressures, whereas the 54% porosity sample exhibited the lowest slope. These results demonstrate that higher porosity enhances compressibility, thereby increasing the connection of conductive pathways under small pressures. This leads to greater resistance changes, significantly improving sensitivity and lowering the detection limit. However, Ma et al. [35] noted that excessively high porosity may result in a fragile porous structure, which can impair the linearity and cyclic stability of the sensor. Therefore, the porosity should be carefully optimized according to the specific application requirements.

Quantum tunneling effect: in terms of band theory, the tunneling conduction mechanism can be described as electron transport across a thin dielectric barrier separating two adjacent conductive fillers. The “gap” between fillers is essentially a dielectric layer, whose thickness determines the tunneling barrier width, while the barrier height corresponds to the energy difference at the metal–dielectric interface. According to Simmons’ model [36], the tunneling current density exponentially depends on both the barrier thickness and barrier height, which explains the pronounced resistance change in piezoresistive sensors under external pressure: compressive strain reduces the barrier width, thus greatly enhancing tunneling probability. The tunneling resistance can be calculated using the following formula:

(3) $$R=\frac{V}{AJ}=\frac{h^{2}d}{Ae^{2}\sqrt{2m\lambda }}\exp\left(\frac{4\pi d}{h}\sqrt{2m\lambda }\right)$$

where *V* is the potential difference, *A* is the cross-sectional area of the tunnel junction, *J* is the tunnel current density, *h* is Planck’s constant, *d* is the distance between adjacent particles, *e* is the electron charge, *m* is the electron mass, and *λ* is the barrier height. This formula indicates that the tunnel resistance is inversely proportional to the cross-sectional area of the tunnel junction and the electron charge, and directly proportional to the distance between adjacent particles.

Regarding how the tunneling effect and connection–disconnection mechanism influence sensing characteristics, the sensors investigated in this study primarily operate through the connection–disconnection mechanism, which generally yields high sensitivity. By contrast, sensors designed for a broader sensing range typically operate via the connection–disconnection mechanism at low pressures—providing high sensitivity—and transition to the tunneling effect at higher pressures, where the sensitivity decreases. This dual-mechanism behavior has also been experimentally verified in porous polymer composites. As shown in Figure 3d, Cai et al. [18] demonstrated that foamed PDMS/CNT sensors exhibited distinct resistance–strain regimes: at small deformations, the sharp resistance drop was dominated by the contact effect of conductive fillers, whereas at larger strains the network stabilized and electron transport relied increasingly on tunneling conduction across narrowed gaps.

## 3. Fabrication Techniques of Porous Structures

### 3.1. Phase Separation

The phase separation method relies on the thermodynamic instability of polymer solutions. By altering the physical or chemical conditions of the system, a homogeneous polymer solution or mixture separates into a polymer-rich phase and a polymer-lean phase. One of the phases is then removed to create a porous structure. Common types of phase separation include non-solvent-induced phase separation (NIPS), polymerization-induced phase separation (PIPS) [37,38,39], polymer blending-phase separation, solvent evaporation-induced phase separation (EIPS), and thermally induced phase separation (TIPS) [40].

**Figure 4:**
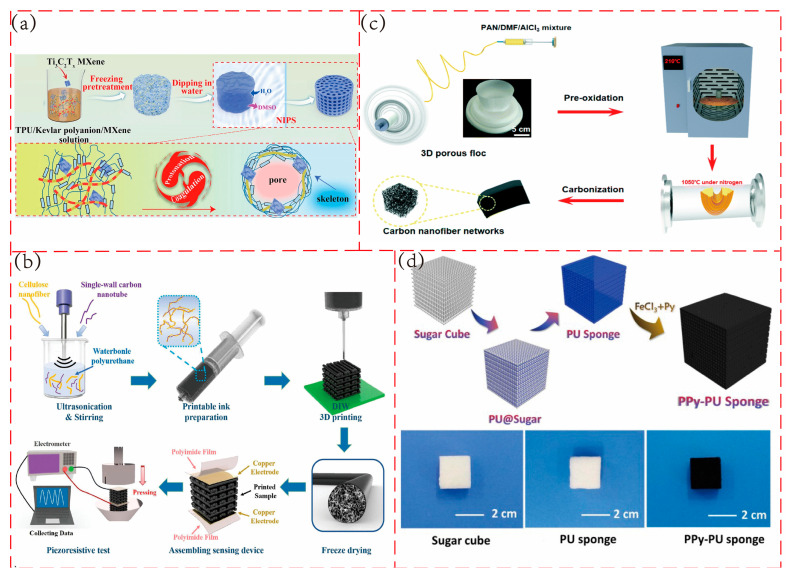
Common fabrication methods of porous structures. (**a**) Phase separation (NIPS method). Adapted with permission from Ref. [41]. Copyright 2023, the authors. (**b**) Three-dimensional printing (DIW method). Adapted with permission from Ref. [48]. Copyright 2024, the authors. (**c**) Electrospinning. Adapted with permission from Ref. [8] copyright 2019, the authors. (**d**) Sacrificial template method. Reprinted with permission from Ref. [26]. Copyright 2020, Elsevier.

In the fabrication of porous polymer flexible piezoresistive sensors, the NIPS method is the most frequently used [4,41]. The NIPS method induces phase separation by immersing the polymer solution in a non-solvent. As shown in Figure 4a, in the preparation of thermoplastic polyurethane/aramid nanofiber/MXene (PAM) foam [41], a mixed solution of TPU, Kevlar polyanion, and Ti_3_C_2_Tx MXene is encapsulated in a dialysis bag, frozen, and then placed in a coagulation bath where deionized water serves as the non-solvent. Water, acting as a proton donor, promotes the conversion of Kevlar polyanion to ANF and simultaneously triggers phase separation. The polymer-rich phase solidifies to form a foam skeleton, while the polymer-lean phase forms the pore structure. This skeleton-pore structure, created through phase separation, combines flexibility with structural support, enabling the sensor to maintain stable resistance responses even after repeated compression. Additionally, methods such as vapor-induced phase separation [42], thermally induced phase separation [43], and polymer blending phase separation [44] are also widely applied in the development of porous polymer piezoresistive sensors.

### 3.2. Three-Dimensional Printing

With the development of 3D printing technology, 3D printing has gradually become an important method for manufacturing porous structures. Three-dimensional printing is a technology that constructs three-dimensional objects by stacking materials layer by layer according to a digital model. It is essentially a layer-by-layer assembly technology, which can accurately control the deposition path and method of materials to form a pore structure. In the preparation of porous polymer piezoresistive sensors, two main methods are used: direct ink writing (DIW) [45,46] 3D printing and fused deposition modeling (FDM) 3D printing [47]. The FDM method generally uses 3D printing to prepare porous molds, which is essentially a sacrificial template method and will not be discussed in depth in this section. The DIW method extrudes ink with appropriate fluidity and viscosity (such as composite ink containing polymers, fillers, etc.) through a device such as a syringe, and deposits it layer by layer according to the designed path to construct a three-dimensional structure.

In practical operations [45,48], computer-aided design (CAD) technology is usually used for 3D modeling of the porous structure to determine the parameters of the pores and the overall structure. Then, appropriate printing materials are selected, and the designed model is imported into a 3D printer. Parameters such as printing speed, temperature, layer height, and filling rate are set for printing. After printing is completed, post-processing such as removing support structures, polishing, and chemical treatment is often required to obtain the ideal porous polymer structure.

3D printing can realize highly complex and personalized design and manufacturing. For example, by adjusting the printing scheme, an anisotropic pressure sensor can be prepared to realize the perception of forces in different directions [48], the manufacturing method is shown in Figure 4b. At the same time, it does not require the production of complex molds, which can shorten the product development cycle and reduce costs, and is suitable for small-batch and customized production. However, the printing speed is relatively slow, which still makes it difficult to meet the needs of large-scale industrial production.

### 3.3. Electrospinning

Electrospinning is a well-established nanofiber production technology. Its fundamental principle involves the formation of a Taylor cone at the nozzle under the influence of a high-voltage electric field. When the electric field force exceeds the surface tension, the polymer solution or melt is ejected, and the solvent volatilizes or solidifies, resulting in the formation of nanofibers. The fiber morphology and pore structure can be controlled by adjusting the electric field parameters, solution properties, and the movement pattern of the receiving device. A porous structure can then be achieved through subsequent treatment processes [49]. As shown in Figure 4c, in the preparation of 3D carbon nanofiber networks [8], a precursor solution is first prepared, then loaded into a syringe. Parameters such as voltage, feed rate, and receiving distance are optimized, and 3D fibrous annular flocs are directly formed on a metal rotating drum covered with vegetable parchment paper. Finally, a 3D porous structure is obtained through a series of post-treatment processes.

Electrospinning offers several advantages. The specifications of the fibers can be precisely controlled by adjusting relevant parameters, allowing the production of fibers with ultra-high porosity, ultra-low density, and high specific surface area, which holds significant application potential. The ultra-high porosity and specific surface area dramatically reduce the detection limit of sensors made from these fibers, enabling them to detect weak pressures (such as pulse fluctuations). This makes electrospun nanofibers particularly valuable for applications in fields like wearable devices, where low detection limits are essential.

### 3.4. Sacrificial Template Method

The sacrificial template method has a long-standing history in the fabrication of porous polymers [50]. The core principle involves using a template with a specific structure to form a polymer around it, and then removing the template to create a porous structure. In the preparation of porous polymers, the template functions as a “mold,” determining the pore shape, size, and distribution of the final porous material. Commonly used templates for fabricating porous polymers include inorganic materials such as polymer nanoparticles and silica nanoparticles, as well as organic compounds like sugar.

In practical applications, sugar particles are favored by researchers due to their cost-effectiveness and the ease of being dissolved and removed with water. As shown in Figure 4d, in the preparation of conductive polypyrrole (PPy)–polyurethane (PU) sponge using the sugar template method [26], a mixed solution of polyurethane prepolymer, chain extenders, and other components is first immersed into the sugar template to allow full penetration of the prepolymer solution into the template’s pore structure. After vacuum treatment to remove the solvent, the sample is cured in an oven at 80 °C for 12 h. The sugar template is then dissolved in deionized water using ultrasonic treatment, resulting in a porous polyurethane sponge, which is followed by PPy polymerization. The pore structure precisely regulated by the template provides a uniform conductive network distribution for the sensor, thereby enhancing its sensitivity and detection stability.

However, it is important to note that the template method has certain limitations. The template material must be dissolved and removed in a suitable solvent bath (such as deionized water), which can be time-consuming and labor-intensive. In some cases, residual template material may remain within the pores, making it difficult to remove and potentially compromising the structural integrity of the final material.

### 3.5. Ice Template Method

The ice template method is a fabrication technique that creates a porous structure through the synergistic effects of low-temperature freezing and vacuum sublimation. It is commonly employed to manufacture aerogel-like flexible piezoresistive sensors. The core principle involves freezing a mixed system containing the polymer matrix, conductive filler, and solvent at low temperatures, causing the solvent to form ice crystals and creating a solid skeleton with a defined shape. This system is then placed in a vacuum environment for an extended period (typically over 12 h), during which the solid solvent is sublimated, leaving behind a three-dimensional network structure composed of the polymer and conductive filler.

For example, in the preparation of a novel aligned porous carbon nanotubes (CNT)/thermoplastic polyurethane (TPU)-conductive foam [51], carbon nanotubes are dispersed in 1,4-dioxane under ultrasonic treatment. TPU particles are added to the suspension, which is stirred at 40 °C until fully dissolved. The mixture is then placed in a directional freezing device containing liquid nitrogen, where the solvent crystallizes into ordered ice crystals through unidirectional heat transfer. After 30 min, the sample is transferred to a freeze dryer and subjected to freeze-drying at ≤5 Pa vacuum and −80 °C for 72 h. The ice crystals are removed, resulting in an aligned porous structure. The ordered arrangement of the pores ensures that the conductive path changes more predictably under pressure, thereby reducing the response time and expanding the linear detection range.

The freeze-drying method offers several advantages: low-temperature treatment helps prevent the degradation of thermosensitive materials, preserving their chemical activity and structural integrity. By adjusting freezing parameters, the anisotropy of the pores can be flexibly designed, allowing researchers to create various types of porous structures [3,51,52]. Furthermore, this method does not require templates or complex chemical reagents, making it more environmentally friendly and reducing the risk of impurity residues. However, a notable disadvantage is that the vacuum sublimation process is generally time-consuming, and maintaining the vacuum freezing state can be energy intensive. Atmospheric pressure drying presents a cost-effective alternative and offers advantages for large-scale production [53].

### 3.6. Microcellular Foaming

Microcellular foaming is a reliable technique for preparing polymer porous structures, commonly utilizing supercritical carbon dioxide (ScCO_2_) as a physical foaming agent [54,55]. Under high pressure and a specific temperature, ScCO_2_ dissolves into the polymer matrix, forming a homogeneous system. By subsequently altering the temperature or pressure, the system enters a supersaturated state, triggering bubble nucleation. As the temperature changes, the bubbles grow within the polymer, eventually forming a porous structure.

The operation of the foaming method is generally straightforward [56,57]. First, the polymer and related additives (such as conductive fillers) are pretreated and dried to remove moisture. The mixture is then exposed to ScCO_2_ under controlled pressure and temperature conditions for a set period, allowing CO_2_ to fully dissolve into the polymer. Once saturation is achieved, rapid depressurization or heating is applied, creating a pressure differential that initiates bubble nucleation and growth, resulting in a porous material. For example, in the preparation of TPU/multi-walled carbon nanotube (MWCNT) composite foam [58], TPU and MWCNT are first mixed to form a solution, which is then placed in a high-pressure vessel, where ScCO_2_ is injected and the mixture is saturated under the set temperature and pressure for a period. Afterward, rapid depressurization and cooling are performed to obtain TPU/MWCNT composite foam with a porous structure. The uniform microporous structure formed by supercritical fluid foaming allows the conductive network in the sensor to change more smoothly under pressure, which helps improve the linear response across a wide pressure range. Sensors based on this composite foam exhibit numerous advantages in pressure detection. The detection range spans from small pressure variations to larger pressure values, making it suitable for various scenarios, including monitoring weak physiological pressures and detecting larger external impacts. Furthermore, the sensors demonstrate exceptional response speed, quickly converting pressure changes into electrical signals and providing real-time feedback on pressure dynamics.

Among various manufacturing routes, the sacrificial template method based on environmentally friendly and low-cost templates (such as salt, sugar, or ice) is particularly promising for scalable and sustainable production. This is not only because this method has a long history and mature technology, but also because the templates can be easily removed by simple water dissolution or sublimation, avoiding the use of harsh chemicals and minimizing environmental impact. Moreover, the widespread availability and low cost of these sacrificial templates make this method highly compatible with industrial-scale manufacturing while allowing for precise control over pore size and distribution.

## 4. Conductive Fillers

Conductive fillers are essential components of porous polymer flexible piezoresistive sensors. Their primary role is to impart stable electrical conductivity to the otherwise non-conductive porous polymer substrate. Without conductive fillers, it is impossible to create an effective conductive path using only the porous structure, and the “pressure-resistance change” signal conversion cannot be achieved, rendering the sensor incapable of performing its sensing function. Common types of conductive fillers include carbon-based materials, polymer conductors, metal conductors, and emerging two-dimensional materials such as MXene. This paper provides an overview of several commonly used conductive fillers. It is important to note that many composite materials utilize more than one type of conductive filler to enhance performance.

### 4.1. Carbon-Based Conductors

Carbon-based conductors constitute a significant category of conductive fillers, primarily including three types: carbon black (CB), carbon nanotubes (CNTs), and reduced graphene oxide (rGO).

CB has been widely used as a conductive filler for porous polymer piezoresistive sensors for an extended period [17,59], typically utilizing nanosized carbon powder. CB offers several notable advantages. As an industrially common material, it is low-cost, readily available, and easy to manufacture. It also exhibits excellent electrical conductivity [3], along with remarkable chemical and thermal stability, which ensures the long-term stable operation of sensors. CB enhances the mechanical properties of the material, and by adjusting its content, it can modify the material’s properties without compromising the polymer’s flexibility [44], making it adaptable to various application scenarios. Notably, the sensor’s performance can be flexibly adjusted by modifying the amount, particle size, surface properties, and composite methods of CB. Simple methods such as the template method [60], ultrasonic treatment [17], and phase separation [44] can be employed to load CB onto the substrate. CB nanoparticles can uniformly fill the small pores in the porous structure, forming dense conductive nodes. For instance, compounding CB into a porous PU substrate significantly improves the sensor’s stability in detecting low-pressure signals [61].

CNTs, a kind of one-dimensional tubular nanomaterial and known for their high electrical conductivity, offer an efficient electron transmission channel. Their high strength and flexibility enhance the mechanical properties of porous polymers, ensuring the sensor’s structural stability under external forces, preventing damage, and improving durability and reliability. The large specific surface area of CNTs not only allows for full interaction with the porous polymer to construct a stable conductive network, but also aids in the adsorption of active substances, further boosting performance. Additionally, CNTs exhibit excellent dispersibility in polymers and can form a uniform conductive path, ensuring consistent response across different regions of the sensor and more stable detection results. Common methods for loading CNTs onto flexible substrates include chemical vapor deposition [62], solution blending [63], freeze-drying [64], and ultrasonic treatment. A special conductive structure can be formed using CNTs to improve sensing performance. For example, a three-dimensional spherical shell-shaped conductive structure can be created through solution blending, endowing the sensor with both piezoresistive and piezocapacitive sensing capabilities, which enhances its sensitivity [65].

Reduced graphene oxide (rGO) features a unique two-dimensional structure, a large specific surface area, and excellent electrical conductivity, which allows it to form a reliable conductive network within porous polymers. Its good flexibility enables the sensor to adapt to various deformations while maintaining stable conductive performance. When compounded with the substrate material, rGO can enhance the mechanical properties of the sensor to some extent and also offers good chemical and thermal stability. These attributes make rGO an excellent material for the preparation of polymer flexible piezoresistive sensors. rGO can be synthesized through various methods [66], typically starting with graphene oxide (GO) and utilizing chemical reduction methods such as hydrazine, catalysts, ascorbic acid [67], and hydroiodic acid. The sensor manufacturing process is relatively simple, with techniques like the dipping method [68] being commonly employed. Graphene sheets can cover the three-dimensional surface of the porous structure, forming a large-area conductive layer. The uniform variation in the contact area of these sheets when the pores are compressed contributes to improved detection accuracy. This property is particularly advantageous in the fabrication of sensors with layered porous structures. For example, when manufacturing an RGO@wood sponge sensor using wood sponge (WS) as the substrate, graphene can be effectively coated onto the lamellar structure of the wood sponge, creating a large-area conductive layer with higher electrical conductivity compared to sensors using other conductive fillers [69].

### 4.2. Polymer Conductors

Polymer conductors have also gained attention in recent studies, with polypyrrole (PPy) and PEDOT:PSS being the most common examples.

PPy is a widely used conductive polymer with a conjugated π-bond structure that provides intrinsic electrical conductivity. The polar groups on its surface can form strong interactions with flexible substrates, allowing for effective integration. The chemical oxidative polymerization method is frequently employed to form a conductive network [26,70], using oxidants like iron ions (e.g., FeCl_3_) to polymerize pyrrole on the substrate surface. PPy can be polymerized in situ within the pores of the porous substrate, aligning well with the pore structure, which ensures that the conductive network responds synchronously with pore deformation, thus enhancing the dynamic performance of the sensor. PPy is biocompatible, making it suitable for designing piezoresistive sensors for wearable devices. By conducting in situ polymerization on the porous flexible substrate, PPy imparts conductivity and can also form microstructures that improve sensor performance. For instance, when PPy is loaded onto the surface of porous PU, it forms a wrinkled structure through in situ polymerization, ensuring a close integration of the conductive layer with the polymer surface and improving detection stability [26].

PEDOT:PSS is a polymer composed of the conductive PEDOT and PSS, which enhances water solubility and processability. With its excellent biocompatibility, good electrical conductivity, flexibility, and solution processability [71,72], PEDOT:PSS has become a common conductive filler for flexible piezoresistive sensors. It can be applied to flexible substrates through processes such as coating and dipping [9]. Its solution easily penetrates the fine pores of the porous structure, forming a uniform conductive path that ensures the sensor maintains stable resistance output even after repeated compression. However, PEDOT:PSS is highly sensitive to humidity and has limited mechanical strength, with its long-term stability in wearable devices requiring further investigation. To enhance the performance of PEDOT:PSS, some studies have explored composites with materials like MXene [73] and carbon nanotubes [74]. PEDOT:PSS also demonstrates great potential when combined with hydrophilic flexible substrates. For example, in the preparation of a PEDOT:PSS@melamine sensor using the dip-coating method, the hydrophilic surface of MS interacts with the aqueous PEDOT:PSS solution, significantly improving the adhesion of the conductive filler. Additionally, molecular forces can further strengthen this bonding property [75].

### 4.3. Metal Conductors

Silver nanowires (AgNWs) are the most common materials among metal conductors, representing nanoscale silver wire structures with a high aspect ratio, similar in shape to nanorods. Their chemical composition is mainly silver elements, and silver atoms are arranged in a specific crystal structure to form nanowires. They are generally obtained by the chemical synthesis of silver salts under specific surfactants and reaction conditions. The current common preparation of silver nanowires mostly adopts the polyol method [76], which can better control the size and aspect ratio of the nanowires. For example, ethylene glycol is used as a solvent and reducing agent, and polyvinylpyrrolidone is used as a surfactant. There are also some other preparation methods, such as the template method and pyrolysis method. Silver nanowires have excellent electrical conductivity and can be used in the manufacture of conductive coatings [77]; there are certain active sites on the surface, which can combine with the substrate material through physical adsorption or chemical bonding, enabling the porous piezoresistive sensor to have good mechanical properties and conductive stability. Silver nanowires can be combined with porous polymers by simple methods, such as the dip-coating method (often used together with the template method to assist in the manufacture of conductive layers) [27], which is very easy to process. The one-dimensional nanowire morphology of AgNWs is highly compatible with the three-dimensional pore structure of porous polymers: on the one hand, the nanowires can flexibly fill the small pores of the substrate and fit closely with the pore walls to form conductive nodes of “line–surface contact”; on the other hand, when the porous structure is deformed under pressure, AgNWs can realize the reversible reconstruction of the conductive path with the compression/rebound of the pores, and the flexibility of the nanowires can avoid structural fracture, ensuring the stability of the conductive network of the sensor during repeated loading-unloading cycles and improving the long-term use reliability.

Liquid metal (LM) has also been used as a conductive filler for porous polymer flexible piezoresistive sensors in recent years and has attracted much attention due to its natural fluidity and high electrical conductivity. It is often compounded with other conductive fillers to enhance the electrical conductivity of the entire conductive layer. For some porous polymers with large pore sizes and relatively dispersed conductive fillers, such as conductive sponges based on carbon nanotubes, the loading of liquid metal can increase conductive nodes and promote the mutual connection of conductive fillers under pressure, thereby improving the conductive performance of the sensor [78].

### 4.4. MXene

MXene is a new type of two-dimensional material, representing layered transition metal carbides or nitrides, with a structure similar to graphene [79]. Its general chemical formula is M_n+1_X_n_T_x_, where M is a transition metal, such as Ti, V, Mo, etc.; X represents carbon and nitrogen elements; T_x_ is a surface functional group, such as -OH, -F, -O, etc., [80,81]. It is generally obtained by chemically etching the MAX phase to remove the A layer (usually main group metals such as Al and Si) [82]. Currently, the commonly used MXene materials mainly use Ti as the transition metal [83,84]. Fluoride-containing etchants are usually used for etching, such as hydrofluoric acid [85], ammonium fluoride, or a mixture of hydrochloric acid and lithium fluoride [86]. There are also some other etching methods, such as electrochemical etching, alkali etching, and molten salt etching [87]. MXene has excellent electrical conductivity [88]; the surface is rich in functional groups, which can be closely combined with the substrate material, enabling the porous piezoresistive sensor to have good mechanical properties. MXene can be attached to the flexible substrate by simple dipping [89] or spraying methods [90]. The two-dimensional ultra-thin sheet morphology of MXene is highly matched with the three-dimensional pore structure of porous polymers: the sheets can flexibly cover the inner walls of the pores to form conductive nodes of “sheet–surface contact”, which greatly increases the conductive interface area and forms a continuous conductive network. When under pressure, the sheets produce significant resistance changes with the deformation of the pores, improving the sensitivity of the sensor.

Table 1 provides a brief description of various conductive fillers.

It should be pointed out that the composite process significantly affects the filler–matrix interfacial quality at the microscopic level. In blending, dispersion of fillers within the polymer matrix is often incomplete, leading to weak or heterogeneous interfaces. Dip-coating deposits a filler-rich layer on the substrate surface, where the bonding is mainly physical adhesion and thus prone to delamination under repeated loading. In contrast, in situ polymerization enables polymer chains to grow directly around the filler surfaces, resulting in stronger chemical or van der Waals interactions and more stable conductive networks. These differences highlight that interfacial engineering is as important as filler type in determining the long-term stability of porous piezoresistive sensors.

## 5. Substrate Materials for Porous Polymer-Based Flexible Piezoresistive Pressure Sensors

### 5.1. Artificial Polymer Materials

#### 5.1.1. Silicone Materials

Silicone is an organic silicone compound whose basic chemical structure is composed of silicon (Si) and oxygen (O) atoms connected by silicon–oxygen chains (Si-O), usually in the form of cross-linking. Silicone materials have good flexibility and stretchability, and their easy processing characteristics make them further become important materials for manufacturing flexible piezoresistive sensors. Recent research on the optimization strategies of piezoresistive sensors based on silicone materials as substrate materials focuses on two directions: structural design [46] and sensitive material optimization [30].

**Figure 5:**
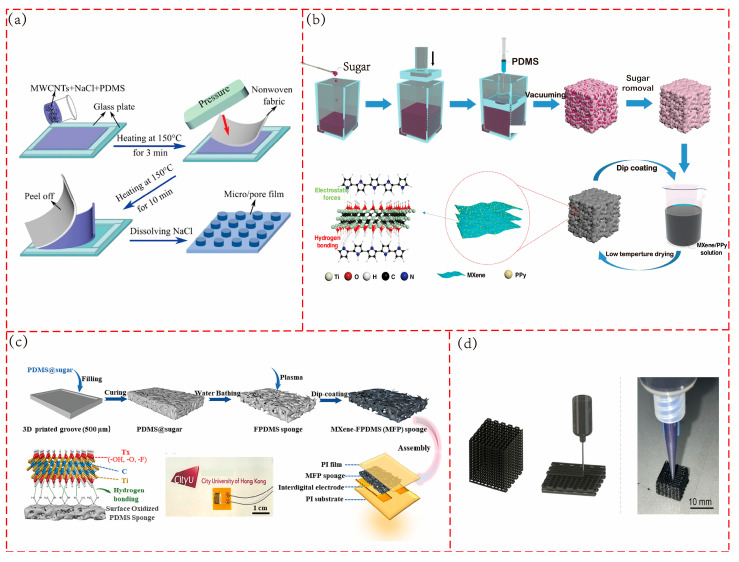
Porous silicone material-based flexible piezoresistive pressure sensors. (**a**) MWCNT/PDMS composites sensor with porous structure and surface microstructure (NaCl template method and nonwoven fabric imprinting). Adapted with permission from Ref. [91]. Copyright 2021, American Chemical Society. (**b**) MXene/PPy@PDMS sponge-based flexible pressure sensor (sugar template method). Adapted with permission from Ref. [92]. Copyright 2023, the authors. (**c**) MXene/FPDMS sponges-based sensor (sugar template method). Adapted with permission from Ref. [30]. Copyright 2024, Elsevier. (**d**) Schematic diagram of fabrication of a soft porous composite flexible pressure sensor (3D printing). Adapted with permission from Ref. [93]. Copyright 2020, American Chemical Society.

In terms of structural design, as shown in Figure 5a, Li’s team [91] developed a flexible piezoresistive sensor based on the combination of porous and surface microstructures. The sensor uses multi-walled carbon nanotube (MWCNTs)/PDMS composite material as the core, and constructs a sensor with both porous structure and surface microstructure through the NaCl template method and non-woven fabric embossing method. The sensor has two linear ranges, with a sensitivity of 10.805 kPa^−1^ in the low-pressure range of 1 Pa–1 kPa and a high sensitivity of 2.015 kPa^−1^ in the range of 1 kPa–10 kPa, which is much higher than that of sensors with ordinary porous structures. The sensor can remain stable after more than 1000 compression cycles under a load of 80 kPa. Qiao et al. [32] developed a flexible piezoresistive sensor based on a carbon nanotube/PDMS composite material with a layered porous structure using a solvothermal method. n-Hexane was used as the solvent, with PDMS prepolymer and crosslinking agent as solutes, carbon nanotubes as conductive fillers, and nickel foam as a template to form the porous structure. The conductivity and elasticity of the sensor were optimized by adjusting the mass fraction of carbon nanotubes and the ratio of PDMS prepolymer to crosslinking agent, respectively. The optimal sensor demonstrated a sensitivity of 0.59 kPa^−1^ in the pressure range of 0.012–260 kPa, a detection limit as low as 12 Pa, a response time of 25 ms, and remained stable after 1700 cycles. In terms of sensitive material optimization, as shown in Figure 5b, Xia’s team [92] developed a flexible pressure sensor based on an MXene/PPy composite material. The sensor was prepared by coating the MXene/PPy composite material onto a PDMS sponge (using sugar cubes as sacrificial templates to form porous structures). Unlike sensors using a single conductive filler, this sensor leveraged the synergistic effect of MXene and PPy, significantly enhancing the performance of the composite material. The sensor exhibited an extremely high sensitivity of 6.8925 kPa^−1^ in the 0–15 kPa pressure range, excellent stability after 5000 compression-release cycles. As shown in Figure 5c, Guo and colleagues [30] developed a flexible piezoresistive sensor based on the Ti_3_C_2_Tx MXene/functionalized PDMS (MXene/FPDMS, further denoted as MPS) sponge, which was prepared by the sugar template method, and after plasma treatment, it was combined with MXene through hydrogen bonds to form an MFP sponge structure, which realized the tight adhesion of the sensitive material to the substrate material and enhanced the strength of the sensor. The sensor has four linear intervals with a sensitivity of 14.2 kPa^−1^ over a low pressure range (0–62 Pa), a detection limit as low as 3.4 Pa, a response time of 90 ms, and stability after 30,000 cycles. As shown in Figure 5d, Tang et al. [93] developed a soft porous composite (SPC) flexible pressure sensor using Ecoflex silicone as the substrate, carbon nanotubes (CNT) as the conductive filler, and fumed silica nanoparticles (SiNPs) as a rheological modifier and insulating filler, fabricated by 3D printing technology. The positive and negative piezoresistive effects of the sensor could be tuned by adjusting the mass ratio of CNT and SiNPs. The optimal SPC-5 sensor exhibited a positive piezoresistive sensitivity of +0.096 kPa^−1^ in the pressure range of 0–175 kPa, with the ability to match high-frequency signals in the range of 0.25–5 Hz, and remained stable after 10,000 cycle tests.

#### 5.1.2. PU

Polyurethane (PU) is a class of polymers synthesized from isocyanates and polyols through an addition reaction. Its basic chemical structure consists of repeated carbamate group units, usually one-dimensional polymer chains. PU foam has excellent mechanical stability [70] and processing performance [1,94], and its inherent three-dimensional porous structure [19] can improve the sensitivity and response speed of the sensor. In addition, PU is easy to process and form, and can be compounded with a variety of conductive materials [20] to further improve the performance of the sensor. PU also has excellent durability and chemical stability [28], and can maintain good performance after multiple cycles of use, which makes it suitable for the manufacture of flexible piezoresistive sensors

**Figure 6:**
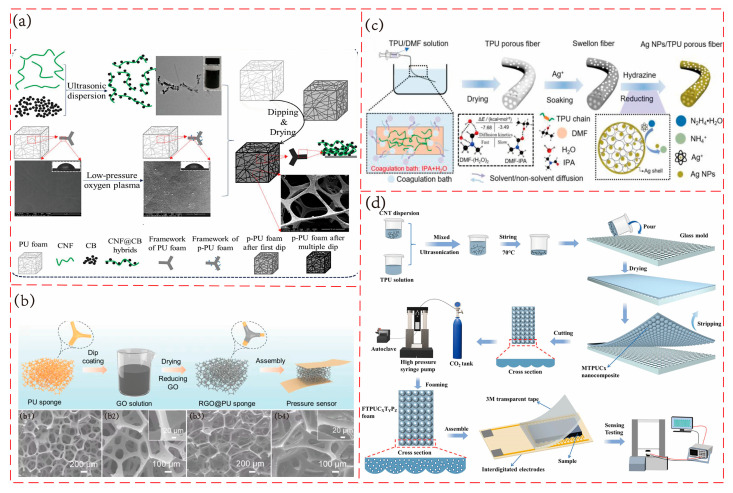
Porous PU-based flexible piezoresistive pressure sensors. (**a**) PU-based flexible piezoresistive sensor based on cellulose nanofiber@carbon black conductive layer. Reprinted with permission from Ref. [95]. Copyright 2020, Elsevier. (**b**) PU/rGO-based flexible piezoresistive sensor; (**b1**,**b2**) SEM images of PU sponge; (**b3**,**b4**): SEM images of rGO@PU.Adapted with permission from Ref. [96]. Copyright 2023, American Chemical Society. (**c**) TPU fiber with uniform porous structure (wet-spinning method). Adapted with permission from Ref. [97]. Copyright 2022, Elsevier. (**d**) TPU/MWCNT composite material with microsphere structure on the surface and porous structure inside (foaming method). Reprinted with permission from Ref. [58]. Copyright 2023, Elsevier.

As shown in Figure 6a, Xu’s team [95] prepared a 3D porous flexible piezoresistive sensor based on cellulose nanofiber (CNF)@carbon black (CB) conductive layer using commercial polyurethane foam as the substrate material. In order to improve the durability of the sensor, the researchers modified and activated the PU scaffold by low-pressure oxygen plasma treatment, introduced oxygen-containing groups into the PU foam, and enhanced the interface interaction between the PU layer and the CNF@CB layer through hydrogen bonds, solving the problems of easy falling off of conductive fillers and general mechanical properties. The sensor has a sensitivity of 0.35 kPa^−1^ in the range of 0–2.2 kPa, good mechanical properties (reaching 29 kPa under 50% compressive strain), and excellent long-term stability. As shown in Figure 6b, Cao’s team [96] developed a flexible piezoresistive sensor based on polyurethane sponge with reduced graphene oxide as the conductive filler. The sensor is constructed by immersing the PU sponge in a graphene oxide solution and performing in situ chemical reduction, which is very simple and economical. The sensor has a high sensitivity of 17.65 kPa^−1^ in the range of 0–3.2 kPa, a wide compressive strain range (0–80%), and shows excellent durability. Thermoplastic polyurethane (TPU) is a type of PU, which has better elasticity, thermal stability, and processing flexibility than traditional PU foam. Using TPU fiber as the substrate material to prepare flexible piezoresistive sensors is also a common preparation method. As shown in Figure 6c, Shi’s team [97] developed a TPU fiber with a uniform porous structure by the wet spinning method and applied it to a piezoresistive pressure sensor. A 30%wt TPU/dimethylformamide (DMF) spinning solution was extruded into a mixed coagulation bath of H_2_O (40%)/isopropyl alcohol (IPA) (60%) to prepare a uniform porous structure. The sensor has small pores with an average pore size of about 3.43 μm. The experimental results show that the fiber with a uniform porous structure has an ultra-high sensitivity of 0.67 kPa^−1^ in the pressure range of 0–624 Pa. As shown in Figure 6d, Huang’s team [58] developed a flexible piezoresistive sensor with a microsphere structure on the surface and a porous structure inside. The sensor is based on TPU and uses the excellent electrical conductivity of multi-walled carbon nanotubes to construct a conductive network. The researchers prepared the TPU/MWCNT composite material using a glass template with a microsphere structure, and then prepared a porous structure using the supercritical carbon dioxide foaming process, which significantly improved its sensing performance. The sensor has a wide working range, with a sensitivity of 0.285 kPa^−1^ in the low-pressure range (0~2 kPa), 0.041 kPa^−1^ in the medium-pressure range (2~10 kPa), and 0.006 kPa^−1^ in the high-pressure range (10~80 kPa). In addition, the sensor can respond quickly and has excellent durability.

#### 5.1.3. PI

Polyimide is a class of polymer materials with high thermal stability and excellent mechanical properties. It is formed by alternating linking of amide bonds (-CO-NH-) and imide bonds (-C=N-). In recent years, polyimide (PI) has become a reliable substrate for preparing porous polymer piezoresistive sensors. It has excellent temperature resistance [23] and can work in a wide temperature range; it has a high modulus [98] and excellent structural stability; it has good chemical stability and easy processing characteristic [99]. In recent years, composite aerogels with PI as the substrate material [100,101,102] have gradually become a hot spot in the manufacture of flexible piezoresistive sensors. Optimizing the distribution of the conductive network and mechanical response through the design of a multi-level pore structure has become a core strategy to improve the sensing performance.

**Figure 7:**
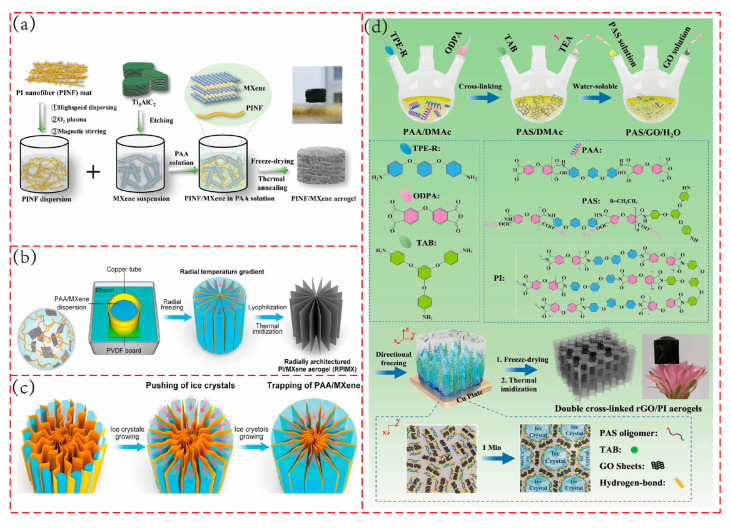
Porous PI-based flexible piezoresistive pressure sensors. (**a**) PINF/MXene aerogel with ordered layered cell structure (freeze-drying and thermal imidization). Adapted with permission from Ref. [103]. Copyright 2021, John Wiley and Sons. (**b**) Polyimide/MXene aerogels with radial lamellar architectures. Adapted with permission from Ref. [104]. Copyright 2022, American Chemical Society. (**c**) Mechanism display of radial structure formed by radial ice template strategy. Adapted with permission from Ref. [104]. Copyright 2022, American Chemical Society. (**d**) Reduced graphene oxide/polyimide nanocomposite aerogel with double cross-linked structure (ice-templating unidirectional solidification method). Reprinted with permission from Ref. [52]. Copyright 2024, Elsevier.

As shown in Figure 7a, Liu et al. [103] prepared a polyimide nanofiber (PINF)/MXene composite aerogel through freeze-drying and thermal imidization processes. The aerogel exhibits an ordered layered cellular structure, with PINF serving as a pillar to support the layered cells. This structure provides the aerogel with excellent compression recovery, similar to that of a spring, with a reversible compression strain of 90%. When used as a piezoresistive sensor, the composite material demonstrates a detection limit as low as 10 Pa, with sensitivity reaching 0.14 kPa^−1^ in the pressure range of 0–5 kPa. It maintains stable performance over 1000 cycle tests (50% strain compression). Notably, the sensor retains its sensing capabilities even at high temperatures up to 150 °C. As shown in Figure 7b,c, Pu’s team [104] fabricated a composite aerogel with polyimide as the substrate and MXene as the conductive filler using an interface enhancement strategy and radial ice template method. By taking advantage of the preferential growth of ice crystals along the radial temperature gradient, a radial porous structure similar to natural wood was formed. This structure provides robust surface support, significantly enhancing the sensor’s pressure resistance. The aerogel has a wide detection range from 60 Pa to 76.5 kPa, achieves rapid response and recovery (response time of 100 ms and recovery time of 80 ms), and exhibits excellent cyclic stability in 1000 cycle tests under 30% strain. As shown in Figure 7d, Xie’s team [52] developed a reduced graphene oxide/polyimide nanocomposite aerogel with a double cross-linked structure for piezoresistive sensors through a combination of ice template unidirectional solidification, freeze-drying, and thermal imidization. The aerogel employs covalent cross-linking of 1,3,5-triaminophenoxybenzene and hydrogen bond cross-linking of graphene oxide to form a unique “porous honeycomb” structure, which imparts high flexibility to the material. This aerogel features a wide detection range (0–85.1 kPa), an short response time, and stable operation across a wide temperature range (from 180 °C to −50 °C). It can still output stable signals after 3000 cycle tests.

### 5.2. Natural Polymer Materials

Compared with artificial polymers, natural polymers have significant advantages in biocompatibility. Their molecular structure is more compatible with biological tissues, which can reduce allergic or rejection reactions when worn by the human body, and are more suitable for close-fitting wearable medical monitoring equipment. At the same time, natural polymers usually have better biodegradability. After the sensor reaches the end of its service life, it can be naturally degraded, reducing environmental pollution and conforming to the development trend of green environmental protection. The following introduces the application of main natural polymer materials such as natural wood, cellulose, and chitosan in polymer porous material flexible piezoresistive sensors.

#### 5.2.1. Cellulose/Natural Wood

Cellulose is primarily composed of glucose molecules that are connected to each other by β-1,4 glycosidic bonds to form long chains. It is the most abundant renewable polymer on Earth [105] and comes from a wide range of sources (wood, cotton, bacteria, etc.). Due to its degradability, non-toxicity, inherent fibrous structure, and the abundance of active groups such as hydroxyl groups on its fiber surface, cellulose easily combines with conductive fillers to form a stable conductive network, enhancing the mechanical strength of composite materials. These properties make it an ideal material for use in wearable flexible piezoresistive sensors.

Natural wood, which is primarily composed of cellulose, lignin, and hemicellulose [2], has high rigidity and is unsuitable as a substrate material on its own. However, after the removal of hemicellulose, it exhibits sufficient flexibility [106]. Coupled with its naturally organized porous structure, it becomes a reliable substrate material for various applications.

**Figure 8:**
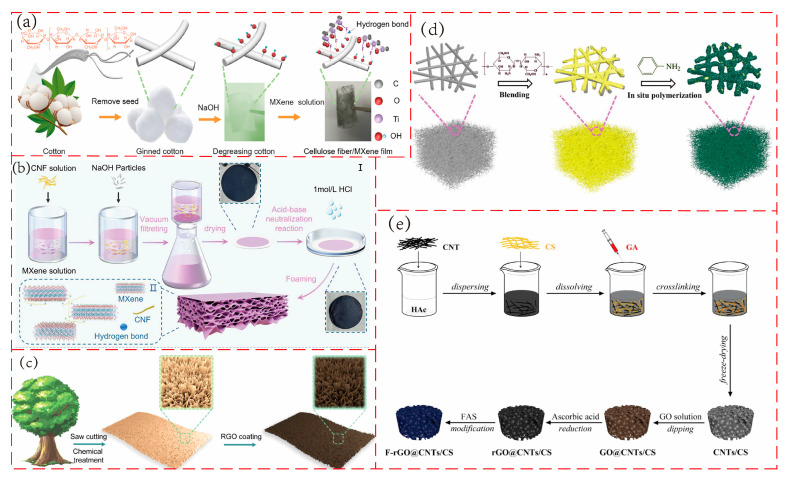
Porous natural polymer-based flexible piezoresistive pressure sensors. (**a**) Cotton fiber-based piezoresistive textile Cellulose fiber/MXene film. Adapted with permission from Ref. [107]. Copyright 2023, Elsevier. (**b**) MXene/cellulose nanofiber composite porous material (acid–base neutralization reaction foaming method). Adapted with permission from Ref. [108]. Copyright 2025, Elsevier. (**c**) Wood-based flexible piezoresistive sensor. Adapted with permission from Ref. [106]. Copyright 2020, American Chemical Society. (**d**) PANI/BC/CH composite aerogel (freeze-drying method). Reprinted with permission from Ref. [109]. Copyright 2019, Elsevier. (**e**) CNTs/CS aerogel (freeze-drying method). Adapted with permission from Ref. [33]. Copyright 2020, Elsevier.

As shown in Figure 8a, Pan and his team [107] prepared a cotton fiber-based piezoresistive textile by attaching MXene sheets to cotton cellulose fibers using the dip-coating method. The strong hydrogen bonds between MXene and cotton fibers enhance adhesion. The sensor demonstrated excellent sensing performance, with a sensitivity of 17.73 kPa^−1^ in the pressure range of 100 Pa–30 kPa, a detection limit as low as 2 Pa, a response/recovery time of 80/40 ms, and remained stable after 5000 cycles. Notably, this material consists of randomly stacked 3D network conductive paths without a specific porous structure. However, more recently, Zhang and colleagues [108] developed a MXene/cellulose nanofiber composite porous material through vacuum filtration combined with an acid-base neutralization reaction. This created a mechanically enhanced structure with surface patchy protrusions and a porous composite. The porous structure effectively increases the spacing between MXene layers, provides rich electron transmission channels, and the compression of the pores under pressure brings the conductive network closer, significantly expanding the resistance change range. The sensor exhibited a sensitivity of 21.457 kPa^−1^, detection range from 0.11 to 11.022 kPa, response/recovery time of 41.84 ms/20.82 ms and stable performance after 6000 cycles. The relevant preparation method is shown in Figure 8b.

As depicted in Figure 8c, Guan and his team [106] utilized natural wood as the material, cutting it vertically to the fiber direction using a circular saw and chopping the surface cell walls to create a ribbon-like rough structure. After chemical treatment to remove hemicellulose and coating with reduced graphene oxide to enhance conductivity, a high-performance wood-based flexible piezoresistive sensor was fabricated. Experimental results demonstrated significant changes in the external current when the ribbon-like rough structure was subjected to pressure, indicating excellent sensitivity. The sensor exhibited a sensitivity of 1.85 kPa^−1^ in a wide linear range of 0–60 kPa, a detection limit of 60 Pa, and remained stable after 10,000 cycles.

#### 5.2.2. Chitosan

Chitosan is a natural polymer polysaccharide obtained after deacetylation of chitin, which is widely found in the exoskeleton of crustaceans (such as shrimp, crabs) and some insects. Its basic chemical structure is a long chain of polysaccharides composed of alternating units of glucosamine and N-acetylglucosamine. Chitosan has long been widely used in the field of biomedicine. In recent years, researchers have increasingly explored its potential as a flexible substrate for porous polymer flexible piezoresistive sensors. However, it is important to note that chitosan itself is relatively brittle and is often compounded with other materials to enhance its mechanical properties.

As shown in Figure 8d, Huang’s team [109] prepared a polyaniline (PANI)/bacterial cellulose (BC)/chitosan (CH) composite aerogel using freeze-drying technology. The aerogel with a BC:CH weight ratio of 1:1 exhibited the best performance as a piezoresistive sensor, with a sensitivity of 1.41 kPa^−1^, a detection limit of 32 Pa, and good stability. The PANI/BC/CH/PDMS composite material, made by embedding the aerogel in polydimethylsiloxane (PDMS), can be used as a strain sensor, capable of detecting various human movements such as finger bending, swallowing, and speech, with significant potential for health-monitoring applications.

As shown in Figure 8e, Wu’s team [33] prepared a carbon nanotube/chitosan (CNTs/CS) aerogel via the freeze-drying method. After dip-coating with graphene oxide (GO), reduction with ascorbic acid, and modification with 1H,1H,2H,2H-perfluorooctyltriethoxysilane (FAS), a conductive and superhydrophobic F-rGO@CNTs/CS aerogel was obtained. The water contact angle of the aerogel reached 154°, and it maintained superhydrophobic stability even under compression. The sensor exhibited a sensitivity of 4.97 kPa^−1^ in the 0–3 kPa range and 0.05 kPa^−1^ in the 40–80 kPa range, with a response time of 170 ms and stable performance after 1000 cycles.

Table 2 summarizes the materials and performance parameters of flexible piezoresistive pressure sensors based on different polymer porous materials reported in recent years.

In addition to the sensing metrics summarized in Table 2, Table 3 provides a qualitative comparison of representative polymer matrices. Mechanical robustness, biocompatibility, and processability are rated using a star system to highlight their relative suitability for wearable and medical applications.

Future research should also address the environmental impact of porous polymer substrates. A key aspect lies in the biodegradability and lifecycle differences between natural and synthetic porous matrices. Natural polymers (e.g., cellulose, chitosan) generally exhibit excellent biodegradability, as they can be enzymatically or hydrolytically degraded into benign byproducts such as glucose or amino acids, thereby offering clear advantages in sustainability. In contrast, synthetic polymers such as PDMS, PU, and PI are far less environmentally friendly. PDMS and PI possess highly stable backbones (Si–O–Si and aromatic imide linkages, respectively), which render them essentially non-biodegradable under natural conditions. PU exhibits slightly better biodegradability due to its urethane bonds, which can undergo partial hydrolysis or enzymatic attack, but its overall degradation rate remains slow compared to natural polymers. Therefore, incorporating quantified assessments of biodegradability and lifecycle analysis is essential for evaluating the environmental footprint of porous substrates, and may guide the rational selection of materials toward more sustainable sensor development.

## 6. Application of Porous Polymer-Based Flexible Piezoresistive Pressure Sensors

In recent years, flexible pressure sensors have made great progress. Thanks to their excellent performance, they have begun to show great application potential in many fields [113,114,115]. As an important part of flexible pressure sensors, flexible piezoresistive sensors based on polymer porous materials have also shown promise in fields such as tactile sensing, biomedical monitoring, and human–computer interaction.

### 6.1. Tactile Sensing

**Figure 9:**
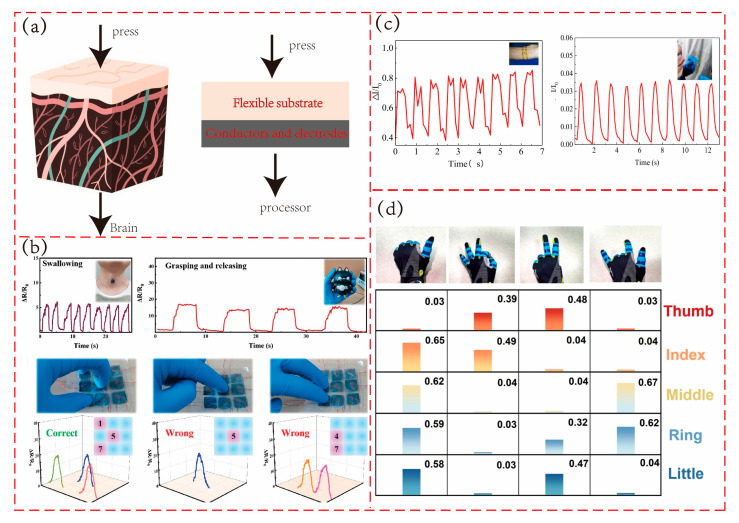
Application of porous polymer piezoresistive sensors. (**a**) Bionic mechanism of electronic skin. (**b**) Application of sensors in the field of tactile sensing. Adapted with permission from Ref. [48] copyright 2024, the authors. (**c**) Application of sensors in biomedical monitoring. Adapted with permission from Ref. [32] copyright 2022, Elsevier. (**d**) Application of sensors in the field of human–computer interaction. Adapted with permission from Ref. [118] copyright 2024, Elsevier.

As a core technology for simulating biological tactile perception of external mechanical stimuli, tactile sensing has strict requirements on the flexibility, sensitivity, and dynamic response ability of sensors in fields such as prosthetic feedback and robotics. A tactile sensor is essentially a bionic device (electronic skin) inspired by human skin. As shown in Figure 9a, for a porous polymer piezoresistive sensor, the flexible substrate is equivalent to the skin surface tissue, responsible for tactile perception, and the conductor and electrode are equivalent to nerve bundles, responsible for transmitting signals to the brain. With the high specific surface area, adjustable mechanical properties, and stable conductive network endowed by its unique three-dimensional porous structure, this type of sensor provides an ideal solution for accurately capturing subtle tactile signals and has become an important support for promoting tactile sensing technology towards bionic-level perception.

Liu et al. [48] used 3D printing to manufacture a waterborne polyurethane (WPU)/single wall carbon nanotubes (SWCNT)/CNF composite sponge for flexible pressure sensing of electronic skin. As shown in Figure 9b, the researchers connected the piezoresistive tactile sensor to the fingertip and tested the tactile performance by holding a toy cat and trying to enter a password. The sensor can quickly output different types of signals in different scenarios, indicating that the sensor can achieve sharp tactile perception. Zhang et al. [116] proposed a porous fluororubber/thermoplastic urethane nanocomposite (PFTNs) for multi-modal tactile sensing. The sensor can not only identify light loads and heavy loads but also identify actions such as pinch, spread, tweak, and torsion.

### 6.2. Biomedical Monitoring

In the field of biomedical monitoring, flexible piezoresistive sensors based on porous polymers have emerged as an ideal solution for accurately capturing human physiological signals due to their excellent flexibility, high sensitivity, and good biocompatibility. Human physiological activities, such as pulse, respiration, and muscle movement, are accompanied by subtle pressure changes. Traditional monitoring equipment often suffers from limitations such as high rigidity and discomfort, while these sensors can conform to the human body, enabling real-time monitoring of physiological signals. They can be integrated into various wearable devices, providing significant support for health management and disease diagnosis.

Pulse, one of the key physiological indicators of the human body, is essential for reflecting overall health status. Qiao and colleagues [32] developed a sensor based on CNT/PDMS composite materials for wrist pulse signal monitoring. As shown in Figure 9c, the sensor was placed on the subject’s wrist to monitor the pulse, with results synchronized with those obtained via electrocardiogram, demonstrating its potential for use in wearable devices for pulse monitoring. Notably, this device can also detect airflow, with the generated signals shown in the second image of Figure 9c, offering a low-cost solution for respiratory monitoring.

Regular physical exercise is crucial for maintaining human health, and monitoring physical activity is essential for effective health management. Wearable devices naturally excel at tracking human motion. Chen and colleagues [117] developed a TPU/polydopamine (PDA)/MXene composite foam piezoresistive sensor for motion monitoring. The sensor can detect plantar pressure distribution, identify abnormal gait, and detect excessive foot pressure, providing timely feedback to encourage corrective walking posture. This sensor shows great potential for applications in intelligent insole design.

### 6.3. Human–Machine Interaction

With the rapid advancement of intelligent technologies such as wearable electronics, smart home systems, and virtual reality, human–machine interaction has evolved into a pivotal link connecting users and digital devices, garnering widespread attention for its potential to reshape how humans engage with technology. A core demand for human–machine interaction is more intuitive, real-time, and high-precision interaction, which relies on sensing components to capture human physical signals (e.g., gestures, vocal vibrations) and convert them into digital information. Thus, flexible piezoresistive pressure sensors based on polymer porous materials have become indispensable in human–machine interaction systems. With high sensitivity to subtle stimuli, rapid response, and good flexibility, they enhance detection accuracy in speech/action recognition, boost interaction naturalness, and advance intelligent human–machine interaction applications.

Yue et al. [118] developed a porous fibrous CNTs/AgNPs/TPU fiber (CATF) that is capable of monitoring various human actions, pronunciation recognition, and gesture recognition. As shown in Figure 9d, a sensor based on this fiber was incorporated into the design of an intelligent glove, which produced different signals corresponding to different gestures of the wearer. Additionally, the sensor can also be utilized for step length recognition and simple speech recognition. Guo et al. [30] prepared a MXene-FPDMS sponge for a high-sensitivity pressure sensor. The sensor quickly recognizes five letters and simple phrases. Notably, the researchers also used a deep learning model to train the sensor, enabling high-accuracy recognition of all 26 English letters, which holds broad application potential in the field of robot speech recognition.

## 7. Summary and Outlook

This paper provides a comprehensive study of flexible piezoresistive sensors based on porous polymers. It reviews various methods for preparing porous structures, such as the sacrificial template method and 3D printing, analyzes their sensing mechanisms, discusses flexible substrate materials such as PDMS and PU, as well as various conductive fillers, and briefly outlines their application areas.

Despite significant advancements in this field, several challenges remain. From a materials perspective, current research primarily focuses on a limited number of porous materials, and there is a need for further development of new porous materials. Additionally, the issue of composite compatibility between conductive fillers and polymer substrates is prominent. Poor interface compatibility leads to unstable conductive networks. Furthermore, temperature-resistant materials are relatively scarce, and most sensors are unable to function reliably in extreme high- or low-temperature environments. In terms of preparation processes, existing methods often face challenges such as high costs and difficulty in scaling up production, which hinder the commercial application of these sensors. Moreover, in practical applications, the stability and reliability of sensors in complex environments still need significant improvement to meet the high precision and stability requirements of sensors used in fields such as intelligent healthcare and industrial monitoring.

For enhancing long-term interface stability and mechanical resilience, future efforts should focus on both interfacial engineering and advanced material design. On the one hand, strategies such as surface functionalization of conductive fillers, incorporation of compatibilizers, and the use of dynamic covalent bonds or supramolecular interactions can strengthen filler–matrix adhesion while allowing stress dissipation during repeated deformation. On the other hand, the introduction of self-healing polymers, double-network structures, and gradient or multilayer architectures may effectively mitigate crack propagation and mechanical fatigue, thereby extending sensor lifetime. Furthermore, hybrid materials that combine robust synthetic polymers with biodegradable or bioinspired components show great promise in reconciling durability with sustainability. These innovations are expected to ensure reliable long-term operation of porous piezoresistive sensors in complex and demanding environments. Looking ahead, the key areas for the future development of flexible piezoresistive sensors based on porous polymers should include the following:

(1) The development of new porous materials, exploring the potential for multi-material composites, and optimizing the composite processes of conductive fillers and substrates. In parallel, future research should also consider the environmental impact of substrate materials, including quantified biodegradability and lifecycle assessments, to better compare natural and synthetic porous polymers and guide the sustainable development of next-generation sensors.
(2) The advancement of low-cost, scalable fabrication technologies to facilitate the translation of basic research into practical applications.
(3) The focus lies on developing composite materials that can maintain relatively stable sensitivity under extreme operating conditions (e.g., extreme temperatures, high fatigue, etc.). Equally important is the establishment of standardized testing protocols and benchmarks to systematically evaluate fatigue and failure modes, which will ensure sensor reliability and enable fair comparison across studies.
(4) The integration of multifunctional sensing capabilities into porous piezoresistive platforms represents another important future direction. Beyond pressure and strain, future designs should aim to incorporate additional modalities such as temperature or humidity into a single architecture. This can be achieved by leveraging hierarchical structures, heterogeneous composites, or hybrid sensing mechanisms, enabling simultaneous and decoupled detection of multiple stimuli. Such multifunctional platforms would significantly broaden the applicability of porous piezoresistive sensors in intelligent healthcare, robotics, and structural monitoring.
(5) Another promising direction is improving signal fidelity and integration. Emerging approaches such as the incorporation of machine learning algorithms for signal processing and pattern recognition, as well as the integration of porous piezoresistive sensors with flexible or miniaturized electronic systems, can enhance data accuracy, reduce noise, and enable real-time intelligent analysis. These strategies will facilitate seamless integration into wearable devices, robotic systems, and industrial monitoring platforms, thereby advancing the practical applicability of porous piezoresistive sensors.

## Figures and Tables

**Table 1:** Brief description of various conductive fillers.

Category	Advantages	Disadvantages	Common Composite Processes
Carbon-Based Conductors	Low cost Excellent chemical stability	Some materials exhibit relatively lower conductivity.	Ultrasonic treatment method, Coating method, Blending method
Polymer Conductors	Good biocompatibility Good flexibility	Poor environmental stability Poor mechanical stability	Dipping method, chemical Polymerization method
Metal Conductors	Good electrical conductivity Easy to process	High cost Easy to oxidize	Dip-coating method
MXene	Excellent electrical conductivity Good mechanical properties	Serious pollution during preparation	Dipping method, Spraying method

**Table 2:** Comparison of performance metrics of flexible piezoresistive pressure sensors based on different polymer porous materials.

Materials	Sensitivity (kPa^−1^)	GF	Pressure Range	Strain Range(%)	Response Time/Recovery Time (ms)	Compression Cycles (Times)	Ref.
CB/PDMS	0.0048 (0–500 kPa)	/	0–500 kPa	/	/	500	[46]
PDMS-based (with PEDOT:PSS conductive layer)	9.51 (0–10 kPa); 0.045 (30–70 kPa); 0.17 (40–120 kPa)	/	0–120 kPa	/	50	1000	[110]
PDMS/CNCs	0.0082 (0–450 kPa)	/	0–450 kPa	/	/	2000	[34]
PINF/MA	22.32 (0–3 kPa); 2.63 (3–8 kPa)	/	0–8 kPa	0.1–50	482/321	1500	[101]
C-PPy@MF	2 (10–90 kPa)	/	10–90 kPa	/	160	5000	[111]
ODA-rGO@PANF/CNTs	33 (0–2 kPa); 4.1 (2–6 kPa)	3.7 (0–17%); 1.418 (18–35%)	/	/	220/140	1000	[112]
GO/PPy@PU	0.79 (0–2.5 kPa)	2.1 (0–40% strain); 0.5 (40–80% strain)	75 Pa–15 kPa	2–85.5	70	10,000	[70]
rGO@PU/ANFs	1.06 (<40 kPa); 2.82(40–58 kPa)	−0.81 (0–56% strain); −5.27 (56–60% strain)	0–58 kPa	0–60	/	10,000	[19]
PU@CNT	51.53 (0.7–3 kPa)	/	0–16 kPa	/	/	8000	[20]
RGO/PI	0.36 (0–4 kPa); 0.01 (4–14 kPa)	/	0–14 kPa	/	80	1000	[23]
PEDOT:PSS/PI	0.021 (0–1 kPa); 0.054 (1–7 kPa); 0.019 (7–17 kPa)	/	0–17 kPa	/	/	200	[99]
MXene/PI	0.83 (0–5.3 kPa); 2.65 (5.3–27.1 kPa)	/	0–27.1 kPa	0–70	/	1000	[102]

**Table 3:** Qualitative comparison of commonly used polymer matrices (★ = lowest, ★★★★★ = highest).

Polymer Matrix	Mechanical Robustness	Biocompatibility	Processability
PDMS	★★★	★★★★	★★★★★
PU	★★★★	★★★★	★★★★
PI	★★★★★	★★★	★★★★
Natural polymers	★★★	★★★★★	★★★

## Data Availability

No new data were created or analyzed in this study. Data sharing is not applicable to this article.
